# Reliability and validity of free‐weight countermovement jumps to characterize force‐velocity‐power profiles

**DOI:** 10.1002/ejsc.12049

**Published:** 2024-03-05

**Authors:** Andrew M. Jonson, Olivier Girard, Bradley A. Wall, Thomas P. Walden, Paul S. R. Goods, Brook Galna, Brendan R. Scott

**Affiliations:** ^1^ The Department of Health and Biostatistics Faculty of Health, Arts and Design Swinburne University of Technology Melbourne Victoria Australia; ^2^ School of Human Sciences (Exercise and Sport Science) The University of Western Australia Perth Western Australia Australia; ^3^ Centre for Healthy Ageing Murdoch University Perth Western Australia Australia; ^4^ KITE Research Institute University Health Network Toronto Ontario Canada; ^5^ Murdoch Applied Sports Science Laboratory Discipline of Exercise Science Murdoch University Perth Western Australia Australia

**Keywords:** field‐based assessment, loaded jumps, lower limb activity, plyometric, Smith machine

## Abstract

The aim of this study was to determine test‐retest reliability and concurrent validity of vertical force‐velocity‐power (FVP) profiles using Smith machine and free‐weight countermovement jumps (CMJs). A repeated‐measure cross‐over design with randomized load order and counterbalanced trials was employed. Sixteen resistance‐trained males (age: 26.4 ± 3.9 years, height: 179.6 ± 8.1 cm, body mass: 84.5 ± 10.8 kg) performed maximal loaded CMJs with 4–50 kg on six occasions, with three trials utilizing a Smith machine and three utilizing free‐weights. Jump height was estimated with a linear‐position transducer, and the Samozino computation method estimated theoretical maximal jump parameters. Reliability and concurrent validity were determined for jump height for each jump load and estimated theoretical maximal jump parameters using estimates of bias (mean difference, 95% limits of agreement) and agreement (intraclass correlation coefficients, ICCs). The jump height and maximum theoretical power demonstrated *good‐to‐excellent* reliability between sessions for both methods (ICC: 0.872–0.947) and concurrent validity between methods (ICC: 0.885–0.969). However, reliability for theoretical maximal force, velocity, and force‐velocity gradient was not as high using either method (ICC = 0.320–0.615) and concurrent validity was *poor* (ICC: 0.122–0.340). In summary, using both jump methods, a linear‐position transducer provides reliable jump height and theoretical maximal power values. However, our data do not support the reliability or validity of FV relationships using linear position transducers.

## Highlights

- Countermovement jump (CMJ) height can be reliably assessed with a linear‐position transducer when adding mass (4–50 kg) with either a Smith machine or free‐weights.
- Theoretical maximum force, velocity, and force‐velocity gradients derived from the Smith machine and free‐weights are neither reliable, nor consistent between methods.
- Smith machine and free‐weight CMJ methods cannot be used interchangeably to develop force‐velocity‐power profiles with a linear position transducer.

## 1 INTRODUCTION

Dynamic strength tests, such as a squat one repetition maximum, are often used to assess lower limb strength (Maio Alves et al., 2010). However, ballistic movements such as vertical jumps may provide more comprehensive information as they independently measure the derivatives of instantaneous neuromuscular performance via maximal power output, which represents the rate at which muscles perform mechanical work (Winter et al., 2016). This measurement demonstrates the underpinning ability to generate high levels of force at a high contraction velocity (Cormie et al., 2011; Samozino et al., 2008). Countermovement jumps (CMJs) are often preferred over squat jumps as they result in greater jump height (Jiménez‐Reyes et al., 2014). This is due to the utilization of the stretch‐shortening cycle, which allows for a greater net impulse (Jiménez‐Reyes, Samozino, Brughelli, & Morin, 2017). While CMJs reflect an athlete's ability to generate force utilizing the stretch‐shortening cycle, force‐velocity‐power (FVP) profiles are developed using a range of loaded vertical jumps. These jumps provide a more comprehensive insight into an individual's lower limb capabilities (Samozino et al., 2012). For instance, with regard to the inverse FV relationship/spectrum, using a range of additional loads while jumping can be useful to determine whether an individual relies more on their ability to generate force or velocity. This determination is based on their ability to produce a higher mechanical impulse against heavy or light loads, representing a “force” or “velocity” reliant FVP profile, in order to produce their maximal power output (Jimenez‐Reyes et al., 2018).

Vertical FVP profiling has gained considerable interest over the last decade to describe the entire lower limb FV spectrum (Samozino et al., 2008). This method typically requires CMJs to be executed with a range of at least five different submaximal loads (ranging between 0 kg and an additional load which allows ∼10 cm jump height) in a Smith machine (Jiménez‐Reyes, Samozino, Pareja‐Blanco, et al., 2017), from which jump height is estimated via an accelerometer (Giroux et al., 2015), a linear‐position transducer (García‐Ramos et al., 2019), or the gold standard force plate application (García‐Ramos, Feriche, et al., 2017). Information about jump height can then be combined with an individual's body mass, leg length in fully extended position, and initial squat depth (i.e., the push‐off position) to compute a vertical FVP profile (Morin & Samozino, 2016) by entering these data into a publicly available spreadsheet (Samozino et al., 2012). From these FVP profiles, the theoretical maximal force (F0), maximal velocity (V0), gradient of the FV relationship (FVgrad), and absolute maximal power (Pmax) can be determined (Jiménez‐Reyes, Samozino, Pareja‐Blanco, et al., 2017). However, the interpretation of whether a FVP profile represents an intrinsic force‐velocity relationship or merely a task‐dependent relationship between effective work and velocity has been recently questioned (Bobbert et al., 2023). Despite its simplicity, this method provides practitioners with valuable insight into lower limb capabilities, which may help prescribe individualized training programs (Jiménez‐Reyes et al., 2019).

Loaded vertical jumps performed using a Smith machine have demonstrated *excellent* reliability in the four key outcome variables (F0, V0, FV‐grad, Pmax) and jump height when using the force plate to develop FVP profiles (Jiménez‐Reyes, Samozino, Brughelli, & Morin, 2017). The *excellent* reliability is potentially aided by smooth vertical displacement of the bar along the fixed rails of the Smith machine, which reduces antero‐posterior movement when performing loaded vertical jumps (Giroux et al., 2015; Jiménez‐Reyes, Samozino, Brughelli, & Morin, 2017). However, inherent limitations are associated with the Smith machine (i.e., high cost, difficulty transporting equipment, restricted maximal jump height), limiting its widespread use within sporting environments. For example, there are anecdotal reports of tall athletes and those with well‐developed jumping capabilities contacting the top of commercially available Smith machines. A more practical method that circumvents these limitations is loaded free‐weight CMJs.

There is increasing popularity with implementing loaded free‐weight CMJs as it is more cost‐effective than a Smith machine and overcomes the limitation of restricting maximal jump height. Unlike a Smith machine, the free‐weight CMJ method allows antero‐posterior and medio‐lateral movements, providing an unrestricted bar path (García‐Ramos, Feriche, et al., 2017), which may allow for a more natural jumping technique. However, greater horizontal bar movement could be expected using free‐weights, which can potentially increase the variability of FVP profiles compared to a Smith machine. To our knowledge, only García‐Ramos, Feriche, et al. (2017) and García‐Ramos, Jaric, et al. (2017) have presented reliable maximal raw force, velocity, and power output variables using a linear‐position transducer for Smith machine and free‐weight CMJs executed against progressive loads (17–75 kg). *Excellent* reliability was demonstrated for both Smith machine (intraclass correlation coefficients [ICCs] ≥0.82; coefficients of variation [CV] ≤6.7%) and free‐weight methods (ICC ≥0.70; ≤7.8%) for maximal force, velocity, and power output variables across all loaded CMJs (García‐Ramos, Jaric, et al., 2017). Although *excellent* reliability was observed for the abovementioned raw values, the validity and reliability of key theoretical FVP profile variables (F0, V0, FVgrad, Pmax) remain unknown when a linear position transducer is used in conjunction with free‐weight CMJs. For practitioners who prescribe lower limb training in their athletes from FVP profiles, it is therefore critical to consider whether both the raw and theoretical variables used to represent these profiles are also reliable.

Therefore, our primary aim was to assess the test‐retest reliability of the jump height for each of the six jump loads (4–50 kg) and the four key theoretical variables (F0, V0, FVgrad, Pmax) commonly used to represent FVP profiles when performing loaded free‐weight CMJs collected with a linear‐position transducer. A secondary aim was to assess the validity of all aforementioned variables using free‐weight FVP profiles compared with the Smith machine FVP profiles. We hypothesized that the free‐weight method would demonstrate reliable jump height and theoretical FVP variables (yet lower than for the Smith machine), and that *excellent* agreement would be observed between methods.

## 2 MATERIALS AND METHODS

### 2.1 Experimental approach to the problem

This study applied a repeated‐measure, cross‐over design to assess test‐retest reliability and concurrent validity for the novel free‐weight CMJ method used to represent vertical FVP profiles. All participants visited the laboratory on seven occasions, each separated by 2–7 days. Participants were familiarized with a range of loaded CMJs (4–50 kg) in their first visit using both the Smith machine and free‐weight methods. Following the familiarization session, participants performed all six experimental trials on separate days, alternating between jump methods (Smith machine or free‐weights) which were counterbalanced to account for any order effect. The jump method for the first trial was randomly assigned to each participant. In each trial, participants completed CMJs with six loads (4, 10, 20, 30, 40, and 50 kg) to develop an individual FVP profile. The inclusion of the 4 kg load represents an unloaded jump (equivalent to the weight of the Smith machine bar), while the heaviest load was selected to enable a jump height slightly exceeding 10 cm (Morin & Samozino, 2016). This study design allowed us to assess the test‐retest reliability of the raw CMJ jump data and the theoretical FVP profile outputs, using both the Smith machine and free‐weight methods. The design also permitted concurrent validity analyses of the free‐weight FVP profiles against the Smith machine FVP profiles.

### 2.2 Participants

A convenience sample of 16 healthy recreationally active males with at least 2 years resistance training history (age: 26.4 ± 3.9 years, height: 179.6 ± 8.1 cm, body mass: 84.5 ± 10.8 kg), but were not regularly performing loaded vertical jumps provided written informed consent to participate in this study. Participants completed a pre‐exercise training history and health screening questionnaire and were excluded if they reported any physical limitations that might compromise testing. They were verbally instructed to avoid any strenuous lower limb exercise 48 h before each testing session. The study was approved by the Institutional Human Research Ethics Committee (2020/104).

### 2.3 Procedures

#### 2.3.1 Familiarization session

During the familiarization session, standing height, body mass, and additional measures related to vertical jumps were initially collected for the Samozino computation method, which incorporates leg length (fully extended foot plantar flexion), initial squat depth (90° knee angle), and push‐off distance (range between leg length and initial squat depth) (Jiménez‐Reyes, Samozino, Pareja‐Blanco, et al., 2017). The participants then performed the standardized warm‐up consisting of 5‐min stationary cycling, dynamic mobility exercises, and preparatory CMJs with a broomstick. Finally, the participants performed at least two maximal effort CMJs against all six trial loads (4, 10, 20, 30, 40, and 50 kg) in incremental order using the counterweighted Smith machine before performing the same jump tasks with free‐weights (2 min rest between jumps). The maximum load of 50 kg was selected as all participants could jump with the correct technique and clear 10 cm in jump height during their familiarization session (Morin & Samozino, 2016; Pérez‐Castilla et al., 2020).

Before each jump, participants were instructed to stand upright with the barbell positioned on their upper *trapezius* using a supportive attachment (Manta Ray, Advanced Fitness Inc.), with both hands firmly gripped over the bar. Once participants were prepared to jump, standardized verbal instructions of “3, 2, 1, jump” were given. Participants then performed a countermovement (knee angle ∼90°) before immediately jumping vertically for maximal height in one continuous movement. A customized string‐line was set to contact the participant's buttocks at 90° knee flexion to ensure appropriate depth. When separation of the bar from the participant's back was noted when jumping, the trial was repeated after a 2‐min rest period.

#### 2.3.2 Loaded vertical jump experimental sessions

Upon arriving for experimental trials, the body mass was gathered, then participants performed the same 10‐min standardized warm‐up as the familiarization session. Subsequently, the Smith machine or free‐weight loaded CMJ exercises were performed against the six trial loads. Loaded CMJs within each trial were performed in randomized order, with the same load sequence maintained for each participant throughout all trials. Two maximal effort CMJ attempts were performed with each load and separated by 2 min of rest and 4 min of recovery between different loads (total session duration ∼45 min). Using a linear‐position transducer device (GymAware, Kinetic Performance Technology), attached to the shaft of the bar inside the right sleeve, jump height was determined as the change in displacement from the starting position (standing erect) to peak positive displacement (maximum jump height). The highest jump for each load was used for subsequent analysis.

The maximal raw force, velocity, and power output during the concentric push‐off phase of each CMJ for all jump loads were calculated using the Samozino computation method (i.e., custom Excel spreadsheet), which incorporates formulas using fundamental laws of dynamics (Asmussen & Bonde‐Petersen, 1974), and three simple variables (i.e., participant body mass + external load, jump height, and push‐off distance) (Samozino et al., 2008). A least‐squares linear regression was used to determine the relationship between the velocity of the participant's center of mass and force generated with each external load + individual's body mass (i.e., individual FV relationship) (Samozino et al., 2014). The intercepts of the FV‐curves, which represent the theoretical maximal production of force and velocity, were extrapolated to obtain F0 (N·kg^−1^) and V0 (m·s^−1^). The FV relationship is calculated as the gradient of the FV slope (FVgrad, in N·s·m·kg^−1^) (Samozino et al., 2012). The Pmax values (W·kg^−1^) were determined as: Pmax = (F0×V0)/4 (Samozino et al., 2008).

### 2.4 Statistical analyses

*Retest reliability* was calculated across the three sessions for free‐weights and Smith machine methods separately. For jump height, reliability was calculated for each load separately. Central tendency and dispersion for each assessment were calculated as the mean and standard deviation. Bias was calculated between session pairs (later session—earlier session) and expressed as mean, standard deviation, 95% limits of agreement [ mean difference ±1.96 (standard deviation of differences)], and the Hedges *g* effect size (adjusting for a small sample size). Additionally, we tested equivalence between sessions assuming an *α* < 0.05 and equivalence region of a Cohen's *d* ± 0.35. ICC estimates across three sessions and their 95% confidence intervals were calculated based on a single‐rating, absolute‐agreement, 2‐way mixed‐effects model (Koo & Li, 2016).

*Concurrent validity* between Smith machine and free‐weight methods was calculated for jump height and FVP profile outcomes. Only data from the first sessions was used for concurrent validity given the adequate reliability of jump height and to better replicate the use of FVP profiles in an applied context. Central tendency and dispersion for each method was calculated as the mean and standard deviation. Bias was calculated as the difference between methods (free‐weights—Smith machine) and expressed as mean, standard deviation, 95% limits of agreement, and Hedges *g* effect size. Equivalence was tested between protocols assuming an *α* < 0.05 and equivalence region of a Cohen's *d* ± 0.35. ICC estimates between the two methods and the corresponding 95% confidence intervals were calculated based on a single‐rating, absolute‐agreement, 2‐way mixed‐effects model (Koo & Li, 2016). Bland and Altman plots were created to visualize the spread of error between methods as a function of the magnitude of measurement (mean of both methods) for each person.

Jump height was the only influential input for the FVP calculations of velocity, force, and power for each load, given body mass and push‐off distance remained similar between sessions. As such, we present reliability and concurrent validity data for jump height and each FVP profile curve characteristic (F0, V0, FVgrad, Pmax, and relative Pmax). Normality of error residuals for each model was checked visually and confirmed with Shapiro–Wilks tests where potential breaches to the assumption of normality were identified. In the absence of practically meaningful differences in FVP profile outcomes, we interpreted ICC values as *poor* (<0.50), *moderate* (0.51–0.75), *good* (0.76–0.90), and *excellent* (>0.91) agreement (Koo & Li, 2016). Similarly, the magnitude of Hedge's *g* effect sizes were interpreted as follows: <0.35 = *trivial*, 0.35–0.80 = *small*, 0.80–1.50 = *moderate*, and >1.50 = *large* (Flanagan, 2013). Analysis was conducted using SPSS statistical package version 29 (SPSS Inc.).

## 3 RESULTS

### 3.1 Reliability

Small to trivial effect sizes indicated there was no bias between sessions for jump height or FVP profile outcomes. Retest reliability between the three sessions was good‐to‐excellent for jump height for both Smith machine and free‐weight protocols, as indicated by ICCs ranging between 0.872 and 0.948 (Table 1). Good‐to‐excellent reliability was also observed for Pmax and relative Pmax derived from FVP profiles (ICC: 0.893–0.945). However, reliability of F0, V0, and FVgrad was poor‐to‐moderate (ICC: 0.320–0.726).

**TABLE 1:** Reliability of countermovement jump height and force‐velocity curve variables across three sessions.

	Mean (SD)			Bias (SD), LoA_95%_			
Condition	Session 1	Session 2	Session 3	Session 2–Session 1	Session 3–Session 1	Session 3–Session 2	ICC (CI_95%_)
Counter movement jump height (mm)							
Free weight							
4 kg	440 (71)	443 (76)	449 (73)	3 (26), *g*: 0.10, LoA: −49 to 54	9 (26), *g*: 0.34, LoA: −42 to 61	6 (25), *g*: 0.25, LoA: −42 to 54	0.938 (0.866, 0.976)
10 kg	419 (64)	424 (64)	422 (66)	5 (17), *g*: 0.31, LoA: −28 to 39	4 (25), *g*: 0.14, LoA: −45 to 53	−2 (21), *g*: −0.09, LoA: −43 to 39	0.947 (0.884, 0.979)
20 kg	388 (56)	397 (66)	384 (54)	9 (24), *g*: 0.37, LoA: −37 to 55	−4 (22), *g*: −0.18, LoA: −47 to 39	−13 (30), *g*: −0.42, LoA: −72 to 46	0.902 (0.792, 0.961)
30 kg	353 (49)	359 (49)	354 (48)	6 (22), *g*: 0.27, LoA: −37 to 49	1 (25), *g*: 0.05, LoA: −47 to 50	−5 (23), *g*: −0.20, LoA: −51 to 41	0.887 (0.765, 0.955)
40 kg	321 (52)	322 (41)	319 (49)	0 (26), *g*: 0.01, LoA: −51 to 51^a^	−2 (22), *g*: −0.10, LoA: −46 to 42	−2 (24), *g*: −0.10, LoA: −48 to 44	0.879 (0.747, 0.952)
50 kg	288 (44)	294 (42)	291 (45)	7 (17), *g*: 0.38, LoA: −27 to 41	4 (23), *g*: 0.16, LoA: −41 to 48	−3 (20), *g*: −0.15, LoA: −43 to 36	0.894 (0.779, 0.958)
Smith machine							
4 kg	490 (63)	496 (65)	500 (66)	6 (30), *g*: 0.19, LoA: −53 to 64	10 (39), *g*: 0.26, LoA: −67 to 87	5 (29), *g*: 0.16, LoA: −52 to 61	0.872 (0.738, 0.949)
10 kg	461 (55)	466 (65)	478 (66)	5 (32), *g*: 0.15, LoA: −58 to 68	17 (36), *g*: 0.47, LoA: −53 to 87	12 (20), *g*: 0.59, LoA: −27 to 51	0.873 (0.735, 0.949)
20 kg	422 (62)	428 (59)	427 (68)	7 (22), *g*: 0.30, LoA: −36 to 49	5 (21), *g*: 0.24, LoA: −36 to 47	−1 (18), *g*: −0.07, LoA: −36 to 34^a^	0.948 (0.888, 0.980)
30 kg	382 (54)	384 (57)	383 (61)	3 (22), *g*: 0.12, LoA: −40 to 45	1 (28), *g*: 0.04, LoA: −53 to 55	−2 (16), *g*: −0.09, LoA: −33 to 30	0.928 (0.843, 0.972)
40 kg	344 (51)	342 (54)	343 (54)	−2 (23), *g*: −0.10, LoA: −48 to 43	−2 (25), *g*: −0.07, LoA: −50 to 47	1 (26), *g*: 0.02, LoA: −50 to 51	0.899 (0.787, 0.960)
50 kg	307 (42)	313 (42)	316 (43)	6 (17), *g*: 0.33, LoA: −27 to 38	8 (18), *g*: 0.45, LoA: −27 to 44	3 (20), *g*: 0.14, LoA: −36 to 42	0.903 (0.796, 0.962)
Force‐velocity curve variables							
Free weight							
F0 (N)	48.3 (12.7)	46.8 (8.6)	45.7 (8.3)	−1.6 (8.9), *g*: −0.17, LoA: −19 to 15.9	−2.6 (8.3), *g*: −0.31, LoA: −18.9 to 13.7	−1.1 (9), *g*: −0.12, LoA: −18.7 to 16.6	0.628 (0.354, 0.833)
V0 (m/s)	2.78 (0.75)	2.77 (0.6)	2.78 (0.51)	0.0 (0.4), *g*: −0.03, LoA: −0.7 to 0.7^a^	0.0 (0.6) *d* < 0.01, LoA: −1.1 to 1.1^a^	0 (0.5), *g*: 0.02, LoA: −0.9 to 0.9^a^	0.726 (0.486, 0.883)
FVgrad (N/m/s)	−19.3 (8.9)	−18.1 (6.7)	−17.3 (5.7)	1.3 (6.4), *g*: 0.19, LoA: −11.2 to 13.8	2 (6.2), *g*: 0.32, LoA: −10.1 to 14.1	0.7 (6.6), *g*: 0.11, LoA: −12.2 to 13.6	0.615 (0.338, 0.826)
Pmax (W)	2693 (435)	2669 (417)	2627 (399)	−25 (157), *g*: −0.15, LoA: −333 to 284	−66 (135), *g*: −0.48, LoA: −330 to 198	−41 (175), *g*: −0.23, LoA: −384 to 301	0.928 (0.845, 0.972)
Pmax (W/kg)	32.1 (6.1)	31.6 (5.4)	31.2 (5.3)	−0.4 (1.9), *g*: −0.22, LoA: −4.1 to 3.2	−0.9 (1.7), g: −0.51, LoA: −4.2 to 2.5	−0.5 (1.8), *g*: −0.24, LoA: −4.1 to 3.2	0.945 (0.880, 0.979)
Smith machine							
F0	44.3 (6.9)	44.1 (9.3)	43 (8.1)	−0.2 (7.2), *g*: −0.02, LoA: −14.4 to 14^a^	−1.3 (7.1), *g*: −0.18, LoA: −15.1 to 12.6	−1.1 (7.6), *g*: −0.14, LoA: −15.9 to 13.7	0.609 (0.326, 0.824)
V0	3.0 (0.3)	3.2 (0.6)	3.3 (0.6)	0.2 (0.6), *g*: 0.31, LoA: −0.9 to 1.3	0.2 (0.7), *g*: 0.36, LoA: −1.1 to 1.6	0.1 (0.6), *g*: 0.11, LoA: −1.2 to 1.3	0.353 (0.054, 0.663)
FVgrad	−15.0 (3.4)	−14.7 (5.4)	−13.8 (4.3)	0.3 (5.2), *g*: 0.05, LoA: −9.9 to 10.4^a^	1.1 (5.2), *g*: 0.21, LoA: −9.1 to 11.4	0.8 (5.2), *g*: 0.16, LoA: −9.4 to 11.1	0.320 (0.010, 0.644)
Pmax	2806 (387)	2897 (447)	2920 (503)	91 (174), *g*: 0.51, LoA: −251 to 433	115 (239), *g*: 0.47, LoA: −353 to 582	24 (167), *g*: 0.14, LoA: −304 to 351	0.893 (0.772, 0.958)
Pmax (W/kg)	33.3 (5.4)	34.5 (6.4)	34.5 (6.0)	1.2 (2.2), *g*: 0.56, LoA: −3.0 to 5.5	1.3 (2.5), *g*: 0.49, LoA: −3.7 to 6.2	0 (1.8), *g*: 0.01, LoA: −3.6 to 3.6	0.922 (0.827, 0.970)

Abbreviations: CI_95%_, 95% confidence intervals; F0, theoretical maximum force; FVgrad, gradient of the force‐velocity curve; *g*, Hedge's *g* effect size; ICC, intraclass correlation; LoA_95%_, 95% limits of agreement; Pmax, maximum power; SD, standard deviation; V0, theoretical maximum force.

^a^ Equivalence between sessions assuming an *α* < 0.05 and equivalence region of a Cohen's *d* ± 0.35.

### 3.2 Concurrent validity

**FIGURE 1:**
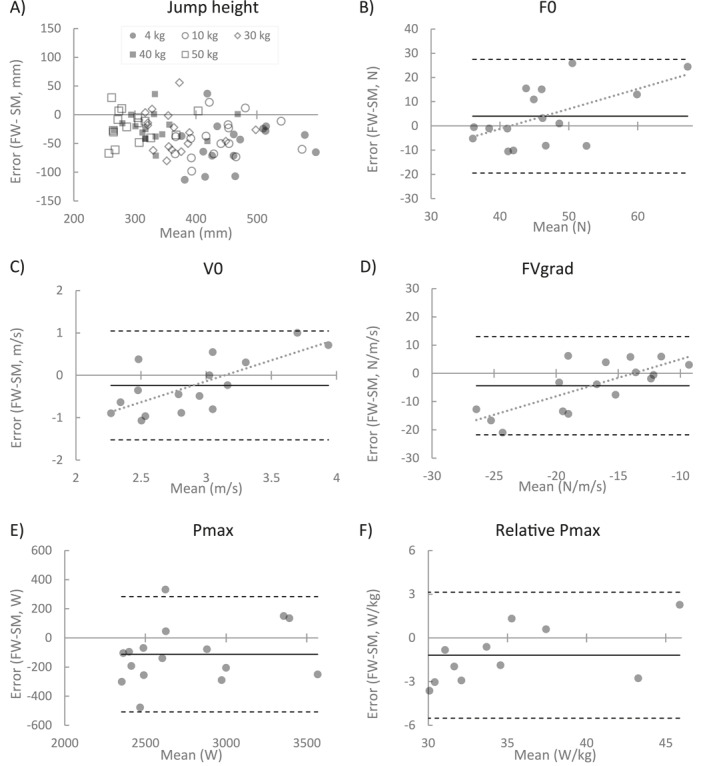
Bland and Altman plots showing the error between FW and SM methods for countermovement jump height (A) and force‐velocity curve variables (B–F). The solid black line shows the mean bias; dashed black lines indicate 95% limits of agreement about the mean bias; lines of best fit are shown by dotted gray lines where there was a significant relationship between the magnitude of measurement and direction of error. Bias and limits of agreement are not shown in panel (A) for each mass and are, instead, reported in Table 2. F0, theoretical maximum force; FVgrad, gradient of the force‐velocity curve; FW, free weight; Pmax, maximum power; SM, Smith machine; V0, theoretical maximum force.

Moderate effects sizes indicate that participants jumped higher when using the Smith machine compared to free‐weights, especially for lower loads, and achieved a higher Pmax (Table 2). The absolute agreement for jump height was moderate‐to‐good (ICC: 0.653–0.802). For variables calculated from FVP profiles, good‐excellent absolute agreement was seen for Pmax(W) (ICC: 0.845) and Pmax(W/kg) (ICC: 0.911). However, agreement was poor for F0, V0, and FVgrad (ICC <0.500). Larger magnitudes of F0 (*r* = 0.573, *p* = 0.020), V0 (*r* = 0.717, *p* = 0.002) and FVgrad (*r* = 0.754, *p* < 0.001) was related to overestimation by the free weights compared to the smith machine (Figure 1). After adjusting for this relationship, there was no correlation between the magnitude of measurement and absolute magnitude of error.

**TABLE 2:** Concurrent validity of jump height and force‐velocity curve variables using free‐weight and Smith‐machine protocols using data from the first assessment.

		Mean (SD)	Free weights–Smith machine	Agreement
Condition	Free weights	Smith machine	Bias (SD), *p*, *d*, LoA_95%_	ICC (CI95%), Pearson's R
Counter movement jump height (mm)				
Free weight				
4 kg	440 (71)	490 (63)	**−50 (39) *p* < 0.001, *g*: −1.213, LoA: −127, 27**	0.653 (−0.061, 0.895), *r* = 0.895
10 kg	419 (64)	461 (55)	**−42 (33) *p* < 0.001, *g*: −1.226, LoA: −106, 22**	0.685 (−0.051, 0.908), *r* = 0.908
20 kg	388 (56)	422 (62)	**−34 (22) *p*:< 0.001, *g*: −1.427, LoA: −78, 10**	0.799 (−0.031, 0.950), *r* = 0.950
30 kg	353 (49)	382 (54)	**−29 (35) *p*: 0.005, *g*: −0.78, LoA: −97, 40**	0.673 (0.138, 0.885), *r* = 0.885
40 kg	321 (52)	344 (51)	**−23 (25) *p*: 0.002, *g*: −0.883, LoA: −72, 26**	0.809 (0.251, 0.942), *r* = 0.942
50 kg	288 (44)	307 (42)	**−20 (27) *p*: 0.011, *g*: −0.688, LoA: −72, 33**	0.739 (0.281, 0.909), *r* = 0.909
Force‐velocity curve characteristics				
F0 (N)	48.3 (12.7)	44.3 (6.9)	4.0 (12.0) *p*: 0.199, *g*: 0.327, LoA: −19.5, 27.5	0.305 (−0.174, 0.680), *r* = 0.680
V0 (m/s)	2.78 (0.75)	3.02 (0.33)	−4.36 (8.86) *p*: 0.068, *g*: −0.48, LoA: −21.73, 13.00	0.122 (−0.285, 0.540), *r* = 0.540
FVgrad (N/m/s)	−19.3 (8.9)	−15.0 (3.4)	−0.2 (0.7) *p*: 0.166, *g*: −0.355, LoA: −1.5, 1.0	0.340 (−0.130, 0.699), *r* = 0.699
Pmax (W)	2693 (435)	2806 (387)	**−113 (202) *p*: 0.042, *g*: −0.542, LoA: −509, 284**	0.854 (0.592, 0.949), *r* = 0.949
Pmax (W/kg)	32.1 (6.1)	33.3 (5.4)	**−1.2 (2.2) *p*: 0.048, *g*: −0.525, LoA: −5.5, 3.1**	0.911 (0.735, 0.969), *r* = 0.969

*Note*: Bold *p* < 0.05 for paired *t*‐test with *df* = 15. Neither jump height nor Force‐Velocity curve characteristics were statistically equivalent between sessions, assuming an *α* < 0.05 and equivalence region of a Cohen’s *d* ± 0.35.

Abbreviations: CI_95%_, 95% confidence intervals; F0, theoretical maximum force; FVgrad, gradient of the force‐velocity curve; *g*, Hedge's corrected effect size; ICC, intraclass correlation; LoA_95%_, 95% limits of agreement; Pmax, maximum power; SG, standard deviation; V0, theoretical maximum force.

## 4 DISCUSSION

The aim of this study was to determine whether the loaded free‐weight CMJs using a linear‐position transducer can provide a reliable and valid method for developing vertical FVP profiles. The Smith machine and free‐weight CMJ methods demonstrated *good‐to‐excellent* reliability for calculating maximal raw force, velocity, power with each jump load, and Pmax (i.e., theoretical absolute maximal power output). However, the Smith machine and free‐weight CMJ methods demonstrated *poor* reliability for F0, V0, and FVgrad (i.e., theoretical FVP profile variables). Our data do not support the determination of FVP profiles using linear position transducers (such as GymAware). In addition, there was a *weak‐moderate* relationship between the Smith machine and free‐weight CMJ derived F0, V0, and FVgrad. The mean jump height was higher and not equivalent when performed with the Smith machine compared to free‐weights. Therefore, we advise against using results derived from these two protocols interchangeably.

Free‐weight CMJs measured with a linear‐position transducer resulted in lower jump height for all jump loads than the Smith machine. However, jump height was reliable for both free‐weight and Smith machine CMJs across all jump loads (ranging from 4 to 50 kg). These reliability findings concur with García‐Ramos, Feriche, et al. (2017) and García‐Ramos, Jaric, et al. (2017) who reported *good‐excellent* reliability for maximal raw force, velocity, and power variables calculated from jump height estimates associated with free‐weight CMJs using a linear‐velocity transducer (ICC: 0.70–0.90, CV: 2.1%–7.8%) and the gold standard force plate (ICC: 0.82–0.97, CV: 1.4%–5.8%). Linear‐position transducers are popular with practitioners due to their simple application for assessing loaded vertical jump performance (Dorrell et al., 2019; Wadhi et al., 2018), despite the Smith machine and force plate traditionally being used when performing loaded vertical jump tasks in laboratory‐based research (Jiménez‐Reyes, Samozino, Pareja‐Blanco, et al., 2017; Samozino et al., 2008). The results from the present study and García‐Ramos, Feriche, et al. (2017) and García‐Ramos, Jaric, et al. (2017) indicate that the easily‐accessible free‐weight CMJs measured using a linear‐position transducer can provide reliable jump height values to represent lower limb vertical jump capabilities, though these values cannot be used interchangeably with those obtained from Smith machine assessments. However, due to limited statistical power, we were unable to demonstrate equivalence between techniques across sessions. Therefore, we cannot definitively state that non‐trivial differences will not occur during testing between sessions.

While the present study demonstrated *good*‐*excellent* reliability in the raw data for all jump loads obtained by both CMJ methods, theoretical F0, V0, and FVgrad variables estimated from the Samozino method exhibited *poor* reliability. These results likely reflect the increased variability in jump performance when it is obtained from a linear position transducer compared to a force place (García‐Ramos, Jaric, et al., 2017). For example, García‐Ramos, Feriche, et al. (2017) and García‐Ramos, Jaric, et al. (2017) demonstrated lower reliability for raw force, velocity, and power values calculated from jump height estimates when measured using a linear‐position transducer (ICC = 0.70–0.95; CV = 2.1%–7.8%) compared with a force plate (ICC = 0.84–0.96; CV = 1.4%–4.9%). Similar findings were also presented by Giroux et al. (2015), where a linear‐position transducer (ICC = 0.86–0.96; CV = 5.0%–12.2%) was shown to be less reliable in measuring maximal raw force, velocity, and power from vertical jumps compared to that obtained by the force plate (ICC = 0.88–0.98; CV = 3.1%–10.6%). It is possible that these discrepancies result from differences in how these technologies measure jumping performance, with a force plate assessing movement based on the center of mass while the linear‐position transducer measures movement of the bar. In addition, it is important to consider that the increased variability associated with the linear‐position transducer during free‐weight loaded vertical jumps may arise from antero‐posterior barbell movement or asymmetry (Lake et al., 2012; McBride et al., 2011). When these raw jump data are extrapolated via the Samozino computation method, as we have examined in the current study, the theoretical F0, V0, and FVgrad values likely demonstrate inflated error due to greater variance associated with the linear position transducer. Considering these collective findings, it seems that the Samozino method may not be appropriate for determining a FVP profile when using data collected with a linear‐position transducer from loaded Smith machine or free‐weight CMJs. However, as we did not use a force plate, future research is needed using this equipment to assess the reliability of theoretical F0, V0, and FVgrad values with both Smith machine and free‐weight CMJs. It should also be acknowledged individualizing training based on FVP profiling does not always improve training adaptations (Lindberg et al., 2021), and more research on the practical applications of this approach is needed.

The Pmax demonstrated *excellent* reliability for both CMJ methods, which contrasts the observations discussed previously for theoretical F0, V0, and FVgrad. Morin and Samozino (2016) have also reported that two individuals can provide equivalent Pmax values while still presenting opposing FV relationships (i.e., force‐vs. velocity‐oriented profile). Therefore, it seems that the high variability associated with the estimated F0 and V0 values does not impact the reliability of Pmax. This notion is supported by García‐Ramos, Feriche, et al. (2017) and García‐Ramos, Jaric, et al. (2017) who reported that Pmax (ICC = 0.93, CV = 3.5%) exhibits higher reliability compared to F0, V0, and FVgrad (ICC = 0.81–0.87, CV = 4.9%–10.5%). The findings presented here also illustrate high variability of F0 and V0 values using a linear position transducer. However, despite the recruitment of individuals who do not perform loaded jumps regularly, Pmax still presented reliable values when collected with a linear position transducer. Whether these results would be replicated in different populations (e.g., females or youth athletes) requires further investigation.

Despite a reliable jump height and Pmax for both jump methods, *poor* agreement for all variables was revealed against the Smith machine for the free‐weight method. In support of these findings, it has been proposed that all force and power output generated by the lower limbs when jumping in a Smith machine is transferred into the vertical displacement of the bar along the fixed rail bar path (García‐Ramos et al., 2015; Pérez‐Castilla et al., 2020). Furthermore, García‐Ramos et al. (2015) and Pérez‐Castilla et al. (2020) indicate overestimation of jump height with the Smith machine due to the constant downward cable tension from the counterbalance weights and low friction forces of the bar along the fixed rail occur when jumping. In support, studies investigating jump capabilities have removed the counterweights and reported similarities between the Smith machine and free‐weights (García‐Ramos, Feriche, et al., 2017; García‐Ramos, Jaric, et al., 2017). Therefore, it could be postulated that the removal of counterweights increases friction forces of the bar along the fixed rail, which reduces vertical displacement when jumping (Pérez‐Castilla et al., 2020). In addition, jump height is one of the key independent variables used to compute vertical FVP profiles (Jiménez‐Reyes, Samozino, Pareja‐Blanco, et al., 2017; Samozino et al., 2012). One can presume equivalent jump height between jump methods (i.e., Smith machine without counterweights and free‐weight) results in similar FVP profiles (García‐Ramos, Feriche, et al., 2017). Pending confirmatory research, this notion could explain why our findings demonstrated *poor* agreement for FV relationship variables (theoretical F0, V0, FVgrad) when comparing a counterbalanced Smith machine to free‐weights.

In summary, loaded CMJs (ranging from 4 to 50 kg) performed in a Smith machine or with free‐weights provide reliable jump height when obtained using a linear position transducer. Due to differences between the Smith machine and free‐weight, one CMJ method should be used consistently. Furthermore, we recommend that the Samozino computation method should not be applied to estimate individuals' vertical FV relationships (i.e., F0, V0, and FVgrad) when data is gathered using a linear‐position transducer with either the Smith machine or free‐weight CMJs due to insufficient reliability. In addition, a more cautious approach to interpreting agreement, namely, using the lower bounds of ICC confidence intervals, further highlights that data collected using a linear‐position transducer should be interpreted with caution when developing individuals' FVP profiles. A larger cohort study is warranted to replicate our findings and provide more precise statistical estimates of agreement. Lastly, when assessing resistance‐trained cohorts (i.e., resistance training experience of ≥2 years) unfamiliar with loaded CMJs, only one familiarization session is required before gathering reliable raw data to characterize lower limb jumping capabilities.

## CONFLICT OF INTEREST STATEMENT

The authors declare no conflicts of interest.
